# Effects of Fermentation with Kombucha Symbiotic Culture of Bacteria and Yeasts on Antioxidant Activities, Bioactive Compounds and Sensory Indicators of *Rhodiola rosea* and *Salvia miltiorrhiza* Beverages

**DOI:** 10.3390/molecules29163809

**Published:** 2024-08-11

**Authors:** Jin Cheng, Dan-Dan Zhou, Ruo-Gu Xiong, Si-Xia Wu, Si-Yu Huang, Adila Saimaiti, Xiao-Yu Xu, Guo-Yi Tang, Hua-Bin Li, Sha Li

**Affiliations:** 1 Guangdong Provincial Key Laboratory of Food, Nutrition and Health, Department of Nutrition, School of Public Health, Sun Yat-Sen University, Guangzhou 510080, China; 2 School of Chinese Medicine, Li Ka Shing Faculty of Medicine, The University of Hong Kong, Hong Kong 999077, China; 3 School of Public Health, Shanghai Jiao Tong University School of Medicine, Shanghai 200025, China

**Keywords:** kombucha, beverage, *Rhodiola rosea*, *Salvia miltiorrhiza*, fermentation, antioxidant activity, polyphenols

## Abstract

Kombucha is a well-known fermented beverage traditionally made from black tea infusion. Recent studies have focused on finding alternative materials to create novel kombucha beverages with various health benefits. In this study, we prepared and evaluated two novel kombucha beverages using *Rhodiola rosea* and *Salvia miltiorrhiza* as materials. The effects of fermentation with the residue of these plants on the kombucha were also investigated. The antioxidant activities, total phenolic contents, and concentrations of the bioactive compounds of the kombucha beverages were determined by the Trolox equivalent antioxidant capacity test, ferric-reducing antioxidant power test, Folin–Ciocalteu method, and high-performance liquid chromatography, respectively. The results revealed that the kombucha beverages made with *Rhodiola rosea* and *Salvia miltiorrhiza* had strong antioxidant capacities and abundant phenolic contents. Additionally, the kombucha fermented with *Rhodiola rosea* residue had higher FRAP, TEAC and TPC values than that fermented without residue. On the other hand, the *Salvia miltiorrhiza* kombucha fermented with residue had similar FRAP and TEAC values but lower TPC values compared to that fermented without residue. The correlation analysis showed that gallic acid, salidroside, and tyrosol were responsible for the antioxidant abilities and total phenolic contents of the *Rhodiola rosea* kombucha, and salvianolic acid A and salvianolic acid B contributed to the antioxidant abilities of the *Salvia miltiorrhiza* kombucha. Furthermore, the kombucha fermented with *Rhodiola rosea* residue had the highest sensory scores among the kombucha beverages studied. These findings suggest that *Rhodiola rosea* and *Salvia miltiorrhiza* are suitable for making novel kombucha beverages with strong antioxidant abilities and abundant phenolic contents, which can be used in preventing and managing oxidative stress-related diseases.

## 1. Introduction

Kombucha is a fermented beverage with a unique sweet and sour flavor, which is traditionally made from black tea (*Camellia sinensis*), sucrose and a symbiotic culture of bacteria and yeasts (SCOBY) [1]. A growing body of studies have found that kombucha has various health benefits, such as antioxidant, anti-inflammatory, antidiabetic, and hepatoprotective activities as well as the ability to regulate gut microbiota [2,3,4,5]. The choice of material used in the preparation of kombucha can significantly impact its bioactive components and bioactivities [6]. As a result, researchers have explored alternative materials (non-*Camellia sinensis*), such as goji berry, oak, and soymilk, for making kombucha [7,8,9]. Furthermore, fermenting kombucha with the residue of plants has been found to enhance the antioxidant capacities and phenolic contents of kombucha beverages made from sweet tea and vine tea in our previous study [10].

*Rhodiola rosea* is a herbaceous perennial plant from the *Crassulaceae* family, which is often cultivated in high-altitude regions. Its rhizome and root have a long history of being used as a traditional medicine and functional food owing to the multiple bioactive compounds present in it, such as salidroside, tyrosol, gallic acid, and epigallocatechin gallate (EGCG). *Rhodiola rosea* possesses antioxidant, anticancer, antidiabetic, hepatoprotective, and neuroprotective activities [11,12,13,14,15]. *Salvia miltiorrhiza* is a member from the *Lamiaceae* family, and has been used as a traditional medicine and functional food. It contains several phenolic compounds, such as danshensu and salvianolic acid, and exhibits antioxidant, anti-inflammatory, anticancer, neuroprotective, and cardiovascular protective effects [16,17,18,19,20,21].

*Rhodiola rosea* and *Salvia miltiorrhiza* are two famous medicinal plants with various bioactivities. However, there is a lack of literature on the production of novel beverages from these plants as materials with kombucha SCOBY fermentation. Moreover, the application of fermented Chinese herbal medicines in the food and medical fields has been a research hotspot, and fermentation usually enhances their antioxidant activities [22]. Therefore, this study aims to develop novel kombucha beverages using *Rhodiola rosea* and *Salvia miltiorrhiza*, which were firstly investigated through kombucha SCOBY fermentation, and to compare the effects of fermentation with or without the residue of plants on the beverage. The analysis will focus on the antioxidant activities, total phenolic contents, concentrations of bioactive compounds, and sensory scores.

## 2. Results and Discussion

**Figure 1:**
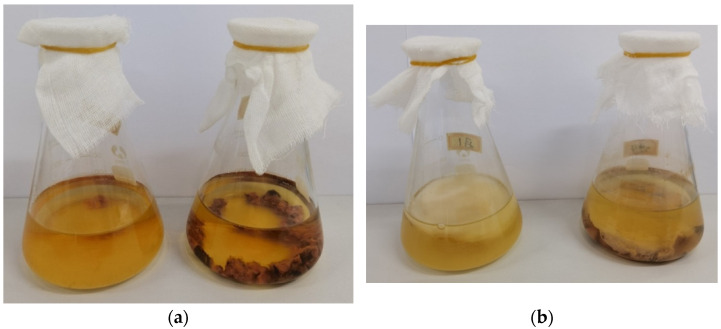
The appearances of kombucha beverages. (**a**) Kombucha fermented without *Rhodiola rosea* residue (**left**) or with *Rhodiola rosea* residue (**right**). (**b**) Kombucha fermented without *Salvia miltiorrhiza* residue (**left**) or with *Salvia miltiorrhiza* residue (**right**).

Kombucha beverages from *Rhodiola rosea* and *Salvia miltiorrhiza* were studied and evaluated for the first time. The appearances of these kombucha beverages are shown in Figure 1. As shown in Figure 1a, the *Rhodiola rosea* kombucha beverages were pale red, and no films were formed, which was different from the other kombucha beverages [10,23]. As shown in Figure 1b, the *Salvia miltiorrhiza* kombucha beverages were yellow and had a thick biofilm.

### 2.1. FRAP Values

**Figure 2:**
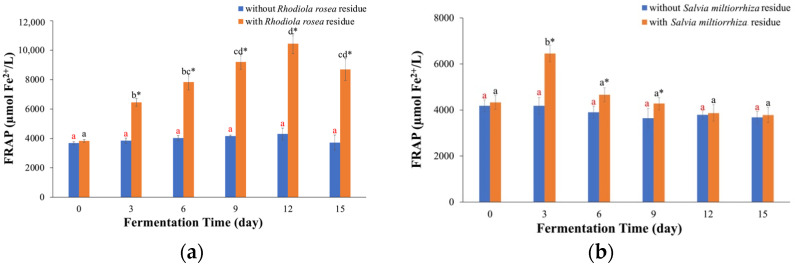
FRAP values. (**a**) Kombucha made from *Rhodiola rosea*, (**b**) kombucha made from *Salvia miltiorrhiza*. The different red letters indicate that there were significant differences among kombucha beverages fermented without residue at different times (*p* < 0.05). The different black letters indicate that there were significant differences among kombucha beverages fermented with residue at different times (*p* < 0.05). * indicates there was a significant difference between fermentation with residue and without residue at the same time (*p* < 0.05).

The FRAP test is used to evaluate the antioxidant activities of substances by reducing Fe^3+^ to Fe^2+^ [24]. For kombucha fermented with *Rhodiola rosea* residue (Figure 2a), the FRAP values increased remarkably as time went on and reached the maximum (10.5 ± 0.7 mmol Fe^2+^/L) on Day 12, and then it decreased. Compared to the FRAP value on Day 0, the FRAP value on Day 12 was up to 2.73 times larger, indicating that fermentation with residue could significantly enhance the antioxidant activities of *Rhodiola rosea* kombucha. For the kombucha fermented without *Rhodiola rosea* residue, the FRAP values remained relatively unchanged. However, when comparing fermentation with and without *Rhodiola rosea* residue, fermentation with residue resulted in higher FRAP values (*p* < 0.05), which were 2.4 times as much as that on Day 12. This finding aligns with those of previous studies, where the FRAP values of fermentation with black tea residue (1.6 times) or green tea residue (3.13 times) were much larger than those of fermentation without residue [23]. This can be attributed to the release of phenolic components from the residue, facilitated by the disruption of the plant cell wall [25].

In the fermentation process of kombucha with *Salvia miltiorrhiza* residue (Figure 2b), the FRAP values initially increased on Day 3 (6.46 ± 0.20 mmol Fe^2+^/L) and then steadily declined. This can be attributed to the greater release of antioxidative compounds from the residue than those from degradation at first, whereas more antioxidative compounds were released from degradation than during the rest of the fermentation process [23]. In contrast, there was no significant change in the FRAP values for fermentation without *Salvia miltiorrhiza* residue. The FRAP values of the kombucha with *Salvia miltiorrhiza* residue were significantly higher on Days 3 (1.5 times), 6 (1.2 times), and 9 (1.2 times) compared to those of fermentation without the residue (*p* < 0.05). On Day 12 and 15, the FRAP values of the kombucha fermented with or without *Salvia miltiorrhiza* residue were similar (*p* > 0.05), and this could be because the reducing substances in kombucha fermented with *Salvia miltiorrhiza* residue were decreased.

### 2.2. TEAC Values

**Figure 3:**
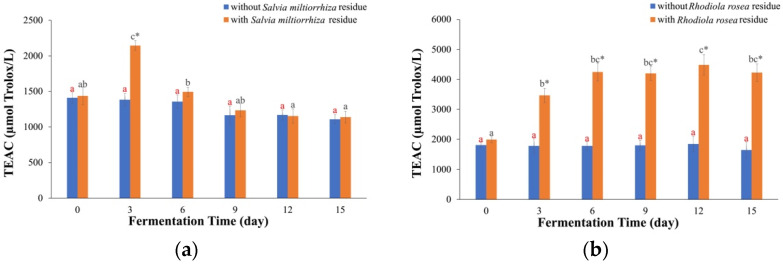
TEAC values. (**a**) Kombucha made from *Rhodiola rosea*, (**b**) kombucha made from *Salvia miltiorrhiza*. The different red letters indicate that there were significant differences among kombucha beverages fermented without residue at different times (*p* < 0.05). The different black letters indicate that there were significant differences among kombucha beverages fermented with residue at different times (*p* < 0.05). * indicates there was a significant difference between fermentation with residue and without residue at the same time (*p* < 0.05).

The TEAC test determines the antioxidant activities of scavenging ABTS^•+^ free radicals [26]. The TEAC values of the kombucha fermented with *Rhodiola rosea* residue (Figure 3a) showed a consistent increase, reaching its maximum (4.48 ± 0.34 mmol Trolox/L) on Day 12 and then remaining stable. The TEAC value on Day 12 was 2.25 times higher than that on Day 0. This indicates that fermentation with residue can enhance the antioxidant capacities of *Rhodiola rosea* kombucha. In contrast, the TEAC values of the kombucha fermented without *Rhodiola rosea* residue remained almost unchanged. Furthermore, fermentation with residue resulted in higher TEAC values compared to fermentation without residue, being 2.43 times greater on Day 12. Similar findings have been reported in previous studies. For example, kombucha prepared with sweet tea residue exhibited a higher TEAC value (1.38 times) compared to that without sweet tea residue [10]. This can be attributed to the fact that the short soaking time of the plant material did not fully extract the compounds from *Rhodiola rosea*, and the compounds in the residue were continuously dissolved into the kombucha beverage through the action of enzymolysis, which could disrupt the plant cell wall [10].

For the kombucha fermented with *Salvia miltiorrhiza* residue (Figure 3b), the TEAC values increased at first and then declined, and the maximum value was 2.14 ± 0.07 mmol Trolox/L on Day 3. For the kombucha fermented without *Salvia miltiorrhiza* residue, the TEAC values were slightly lowered. On Day 3 only, the kombucha with *Salvia miltiorrhiza* residue had a higher TEAC value (1.55 times) than that without *Salvia miltiorrhiza* residue. In total, the change trend and reason for the TEAC values of the *Salvia miltiorrhiza* kombucha were similar to those of its FRAP values.

### 2.3. TPC Values

**Figure 4:**
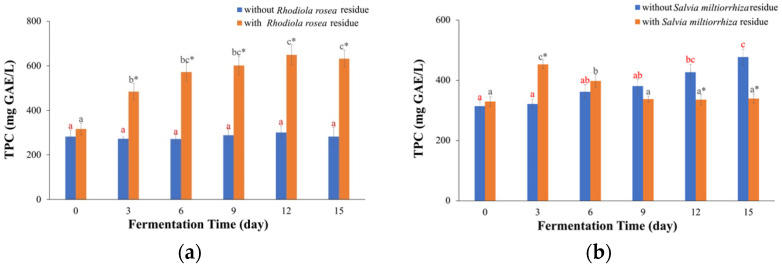
TPC values. (**a**) Kombucha made from *Rhodiola rosea*, (**b**) kombucha made from *Salvia miltiorrhiza*. The different red letters indicate that there were significant differences among kombucha beverages fermented without residue at different times (*p* < 0.05). The different black letters indicate that there were significant differences among kombucha beverages fermented with residue at different times (*p* < 0.05). * indicates there was a significant difference between fermentation with residue and without residue at the same time (*p* < 0.05).

For kombucha fermented with *Rhodiola rosea* residue (Figure 4a), the TPC values increased until Day 12 and then remained stable, consistent with the results of the FRAP and TEAC values. The highest TPC value was 0.649 ± 0.046 g GAE/L on Day 12, which was 2.05 times that of Day 0. For the kombucha fermented without *Rhodiola rosea* residue, there were no significant differences in the TPC values at different time points. However, when compared to the kombucha fermented without residue, the kombucha fermented with residue had higher TPC values on Day 3 (1.78 times), 6 (2.11 times), 9 (2.08 times), 12 (2.16 times) and 15 (2.24 times). Based on the FRAP, TEAC, and TPC results, the kombucha fermented with *Rhodiola rosea* residue had stronger antioxidant abilities and more phenolic compounds. Therefore, it can be inferred that fermentation with *Rhodiola rosea* residue made fuller use of the antioxidative compounds present in *Rhodiola rosea*, which makes it suitable for producing beverages with better health benefits.

For the kombucha fermented with *Salvia miltiorrhiza* residue (Figure 4b), the TPC values initially increased and then decreased, similar to the trends observed in the FRAP and TEAC values. The highest TPC value was 0.453 ± 0.015 g GAE/L on Day 3, which was 1.38 times that on Day 0. This can be attributed to the release of phenolic compounds from the residue. In contrast, the kombucha fermented without *Salvia miltiorrhiza* residue showed a constant increase in TPC values with fermentation time, which differed from the results of the FRAP and TEAC tests. The maximum value was 0.477 ± 0.027 g GAE/L on Day 15. This upward trend might be due to the decomposition and structure modification of some complex phenolic compounds into easily detectable phenolic compounds as time goes on, thereby increasing the TPC values of kombucha [27,28]. However, it should be noted that the increase in the TPC values alone does not necessarily indicate an increase in the total antioxidant capacities of kombucha, as the total antioxidant capacities can also be influenced by antagonism and synergy between phenolic compounds and other compounds present in kombucha [25].

### 2.4. Bioactive Compounds in New Kombucha

**Figure 5:**
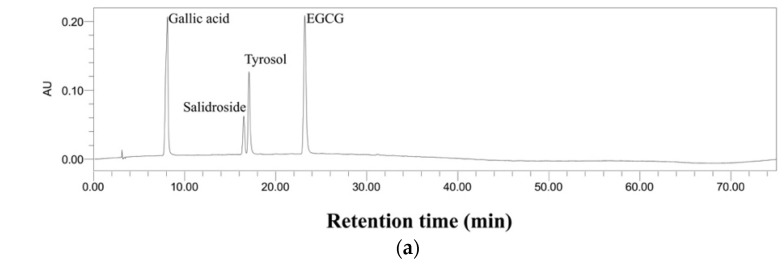

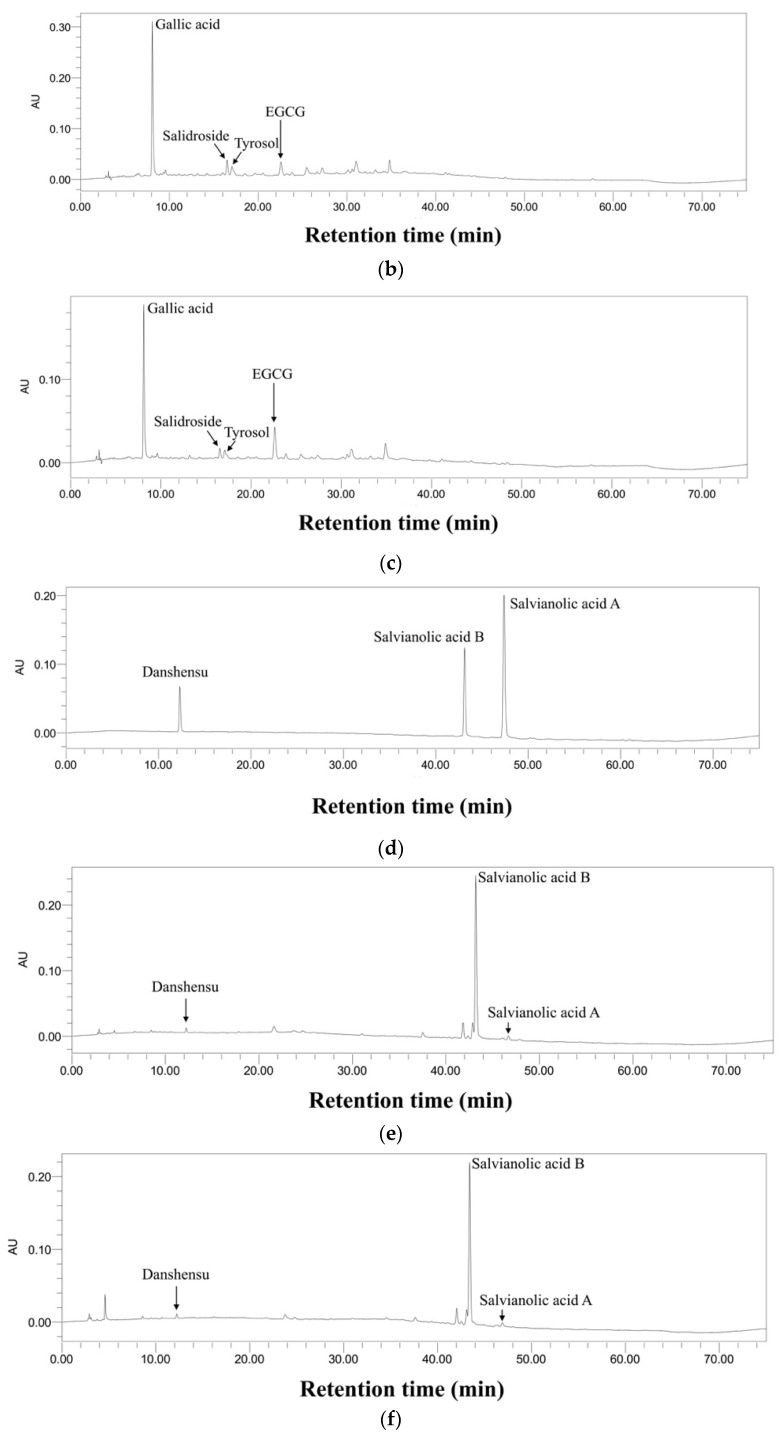
Chromatograms of standards and kombucha beverages. (**a**) Standards for *Rhodiola rosea* at 275 nm, (**b**) kombucha fermented with *Rhodiola rosea* residue at 275 nm, (**c**) kombucha fermented without *Rhodiola rosea* residue at 275 nm, (**d**) standards for *Salvia miltiorrhiza* at 287 nm, (**e**) kombucha fermented with *Salvia miltiorrhiza* residue at 287 nm, (**f**) kombucha fermented without *Salvia miltiorrhiza* residue at 287 nm. EGCG, epigallocatechin gallate.

As shown in Figure 5, several bioactive compounds in the kombucha beverages were identified by comparing the retention time and UV–visible spectra with those of the standard compounds. The standard compounds are displayed in Figure 5a,d. The gallic acid, salidroside, tyrosol, and EGCG were identified in the kombucha beverages from *Rhodiola rosea* (Figure 5b,c), and danshensu, salvianolic acid A, and salvianolic acid B were determined in the kombucha beverages from *Salvia miltiorrhiza* (Figure 5e,f). The concentrations of the compounds in the kombucha beverages fermented with *Rhodiola rosea* or *Salvia miltiorrhiza* are shown in Table 1 and Table 2.

**Figure 6:**
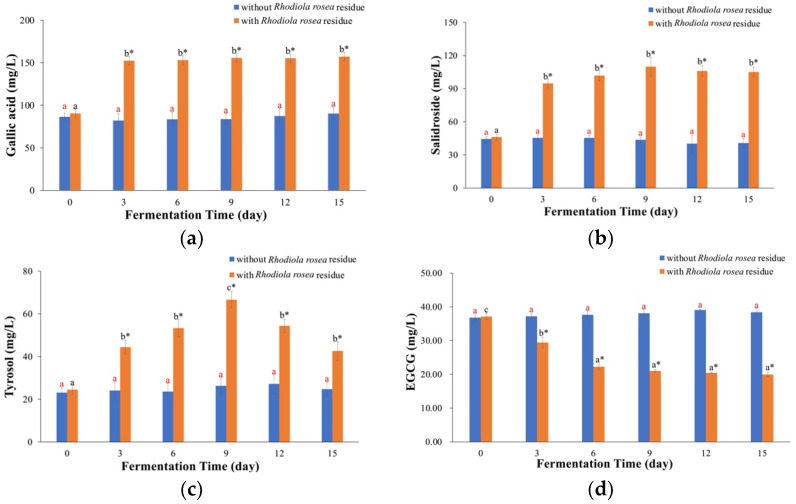

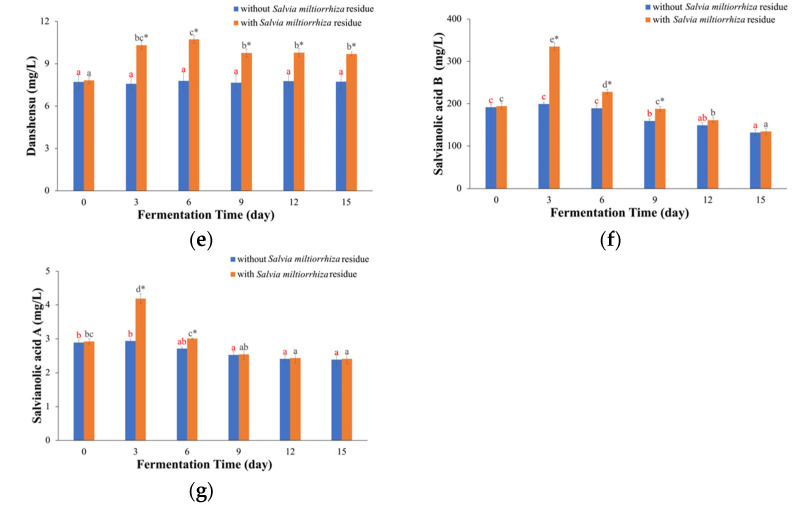
The concentrations of bioactive components in kombucha beverages. (**a**–**d**) *Rhodiola rosea* kombucha, (**e**–**g**) *Salvia miltiorrhiza* kombucha. The different red letters indicate that there were significant differences among kombucha beverages fermented without residue at different times (*p* < 0.05). The different black letters indicate that there were significant differences among kombucha beverages fermented with residue at different times (*p* < 0.05). * indicates there was a significant difference between fermentation with residue and without residue at the same time (*p* < 0.05).

For the kombucha fermented with *Rhodiola rosea* residue, the concentrations of gallic acid (Figure 6a) and salidroside (Figure 6b) sharply increased on Day 3 and then maintained a high level for the remaining fermentation time. The increase in gallic acid and salidroside might be attributed to the dissolution from *Rhodiola rosea* residue. Moreover, the concentration of tyrosol increased until Day 9 (2.71 times compared to Day 0) and declined after that (Figure 6c). The reason for this might be that tyrosol could be continually released from the residue, but then the enzymatic degradation of tyrosol into other compounds could be stronger. However, EGCG consistently declined over time (Figure 6d), which could be attributed to the enzymes in kombucha transforming EGCG into other bioactive compounds [27,29,30]. On the other hand, the concentrations of these four phenolic compounds did not significantly change as the fermentation time increased in the kombucha fermented without *Rhodiola rosea* residue.

In the case of the kombucha fermented with *Salvia miltiorrhiza* residue, the concentration of danshensu (Figure 6e) initially increased and then slightly decreased. The concentration of danshensu on Day 15 was still higher than that on Day 0 (*p* < 0.05). Additionally, the concentrations of salvianolic acid B (Figure 6f) and salvianolic acid A (Figure 6g) increased on Day 3 and then sharply declined. This change trend was consistent with those of the FRAP, TEAC and TPC values in the kombucha fermented with *Salvia miltiorrhiza* residue. Compared to the concentrations of salvianolic acid B and salvianolic acid A on Day 0, those on Day 3 were up to 334.77 ± 8.91 mg/L (1.72 times) and 4.19 ± 0.14 mg/L (1.43 times), respectively (*p* < 0.05). When the release from the residue was stronger than that from degradation, the concentrations of these compounds increased. However, when the degradation was stronger afterwards, the concentrations decreased [10]. For the kombucha fermented without *Salvia miltiorrhiza* residue, the concentration of danshensu remained almost unchanged, but the concentrations of salvianolic acid B and salvianolic acid A showed a decreasing trend, which could be because of the degradation.

The bioactive compounds in the kombucha beverages made from *Rhodiola rosea* and *Salvia miltiorrhiza* were quite different from those in traditional kombucha beverages made from *Camellia sinensis* [31,32]. The differences were because the bioactive compounds of the kombucha beverages could be largely affected by the fermented material [6]. The salidroside, tyrosol, danshensu, salvianolic acid B, and salvianolic acid A in the kombucha beverages from *Rhodiola rosea* and *Salvia miltiorrhiza* could provide beneficial effects to these kombucha beverages, such as preventing neurodegenerative diseases, diabetes mellitus, hypoxic pulmonary hypertension, and altitude sickness [33,34,35,36], which should be verified in future studies. Moreover, these kombucha beverages did not contain caffeine, which was found in a high concentration in traditional kombucha made from *Camellia sinensis* [23]. Therefore, kombucha beverages from *Rhodiola rosea* and *Salvia miltiorrhiza* could be better choices for caffeine-intolerant people as well as the pregnant and children [37].

### 2.5. Correlation between Antioxidant Parameters and Compounds

**Figure 7:**
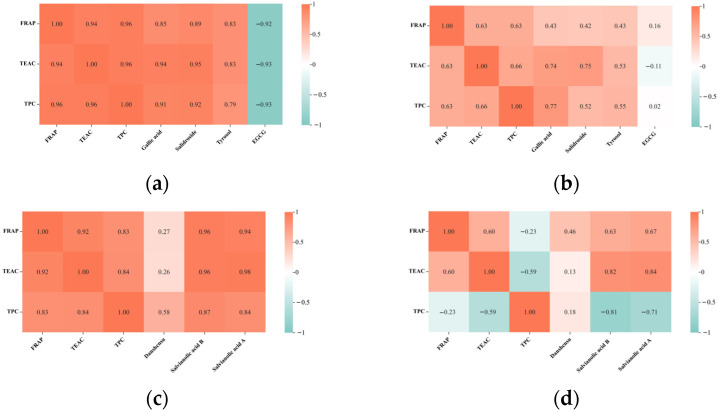
Heatmaps of parameters and compound concentrations. (**a**) Kombucha fermented with *Rhodiola rosea* residue, (**b**) kombucha fermented without *Rhodiola rosea* residue, (**c**) kombucha fermented with *Salvia miltiorrhiza* residue, (**d**) kombucha fermented without *Salvia miltiorrhiza* residue. EGCG, epigallocatechin gallate. The red color means positive correlation, and the blue color means negative correlation. The darker the color, the stronger correlation.

As shown in Figure 7, for the FRAP and TEAC values, they displayed significant correlations in the *Rhodiola rosea* kombucha beverages (with residue: R = 0.94; without residue: R = 0.63) and *Salvia miltiorrhiza* kombucha beverages (with residue: R = 0.92; without residue: R = 0.60), indicating that the bioactive compounds in these kombucha beverages possessed both a reducing power and the ability to scavenge free radicals. For the FRAP and TPC values, the correlations were significant in the *Rhodiola rosea* kombucha (with residue: R = 0.96; without residue: R = 0.63) and *Salvia miltiorrhiza* kombucha with residue (R = 0.83), suggesting that the phenolic compounds could be one of the contributors to the reducing power of these kombucha beverages. For the TEAC and TPC values, there were correlations found in the *Rhodiola rosea* kombucha (with residue: R = 0.96; without residue: R = 0.66) and *Salvia miltiorrhiza* kombucha with residue (R = 0.84), which indicated that the phenolic compounds could be one of the contributors to the free radical-scavenging ability of these kombucha beverages.

Concerning the correlation between parameters and the concentration of bioactive compounds in the kombucha made from *Rhodiola rosea* (Figure 7a,b), (1) there were strong correlations between the FRAP values and gallic acid (R = 0.85) and salidroside (R = 0.89) as well as tyrosol (R = 0.83) in the kombucha fermented with residue. (2) Relationships were found between the TEAC values and gallic acid (with residue: R = 0.94; without residue: R = 0.74) and salidroside (with residue: R = 0.95; without residue: R = 0.75) as well as tyrosol (with residue: R = 0.83) in the kombucha. (3) There were relationships between the TPC values and gallic acid (with residue: R = 0.91; without residue: R = 0.77) and salidroside (with residue: R = 0.92) as well as tyrosol (with residue: R = 0.79) in the kombucha. These correlations illustrated that gallic acid, salidroside, and tyrosol were responsible for the reducing power, free radical-scavenging ability and total phenolic contents of the *Rhodiola rosea* kombucha.

With regard to the correlation between the parameters and bioactive components in the *Salvia miltiorrhiza* kombucha (Figure 7c,d): (1) there were correlations between the FRAP values and salvianolic acid B (with residue: R = 0.96; without residue: R = 0.63) as well as salvianolic acid A (with residue: R = 0.94; without residue: R = 0.67) in the kombucha. (2) Relationships were found between the TEAC values and salvianolic acid B (with residue: R = 0.96; without residue: R = 0.82) as well as salvianolic acid A (with residue: R = 0.98; without residue: R = 0.84) in the kombucha. (3) Relationships were found between the TPC values and salvianolic acid B (R = 0.87) as well as salvianolic acid A (R = 0.84) in the kombucha fermented with residue. These correlations elucidated that salvianolic acid B and salvianolic acid A may contribute to the reducing power and scavenging free radical ability of the *Salvia miltiorrhiza* kombucha. Moreover, salvianolic acid B and salvianolic acid A were involved in the TPC values of the kombucha fermented with *Salvia miltiorrhiza* residue.

### 2.6. Sensory Scores of Kombucha

**Figure 8:**
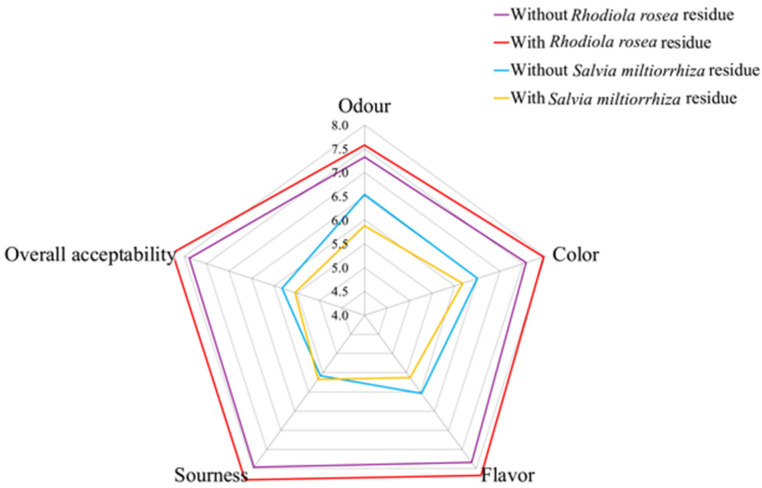
The sensory analysis results of kombucha beverages from *Rhodiola rosea* and *Salvia miltiorrhiza*.

The sensory results of these kombuchas are shown in Figure 8. The scores of odor, color, flavor, sourness, and overall acceptability were slightly higher in the kombucha fermented with *Rhodiola rosea* residue compared to the kombucha fermented without *Rhodiola rosea* residue, although the differences were not significant (*p* > 0.05). Similarly, there were no significant differences (*p* > 0.05) in the scores of the five parameters between the *Salvia miltiorrhiza* kombucha beverages fermented with and without residue. Additionally, the *Rhodiola rosea* kombucha received higher sensory scores compared to the *Salvia miltiorrhiza* kombucha. Overall, fermentation with residue can enhance the exploration of antioxidative compounds in the raw material and slightly improve the sensory characteristics of *Rhodiola rosea* kombucha. Furthermore, fermentation with residue did not affect the sensory properties of the *Salvia miltiorrhiza* kombucha.

## 3. Materials and Methods

### 3.1. Materials

The dried roots of *Rhodiola rosea* and *Salvia miltiorrhiza* were purchased from a branch of Tongrentang company (Guangzhou, China). They are not threatened species, and can be traded freely in China. The kombucha SCOBY contains a symbiotic culture of yeasts, acetic acid bacteria, and lactic acid bacteria, such as *Zygosaccharomyces*, *Gluconacetobacter*, and *Lactobacillus*, and was obtained from Shandong Ruyun Edible Fungus Planting Co., Ltd. (Liaocheng, China).

Hydrochloric acid, K_2_S_2_O_8_, FeSO_4_·7H_2_O, FeCl_3_·6H_2_O, sodium acetate, and acetic acid were purchased from Tianjin Chemical Factory (Tianjin, China). The Folin–Ciocalteu phenol reagent, 6-hydroxy-2,5,7,8-tetramethylchromane-2-carboxylic acid (Trolox), 2,2′-azinobis (3-ethylbenothiazoline-6-sulfonic acid) diammonium salt (ABTS), gallic acid, and 2,4,6-tri(2-pyridyl)-S-triazine (TPTZ) were obtained from Sigma-Aldrich (St. Louis, MO, USA). Formic acid, sucrose, and methanol were bought from Macklin Chemical Factory (Shanghai, China). Na_2_CO_3_ was obtained from Shanghai Yuanye Biological Technology Co., LTD. (Shanghai, China). The standard compounds EGCG, gallic acid, salidroside, tyrosol, danshensu, salvianolic acid A, and salvianolic acid B were bought from Derick Biotechnology Co., Ltd. (Chengdu, China).

### 3.2. Preparation of Kombucha Beverages from Rhodiola rosea and Salvia miltiorrhiza

Kombucha SCOBY was activated according to the methods of the previous study [23]. Kombucha beverages from *Rhodiola rosea* and *Salvia miltiorrhiza* were prepared as follows: 200 mL distilled water and 20 g sugar were added into a flask, which was heated in a water bath pot and stirred constantly to dissolve the sugar. When the temperature of the pot reached 100 °C, 2 g of *Rhodiola rosea* or *Salvia miltiorrhiza* were added and the mixture was boiled for 5 min. After cooling to room temperature (25 °C), the infusion was filtered into another flask for further fermentation without residue, while fermentation with residue was conducted without filtration. Subsequently, 20 mL of activated kombucha starter culture was put into the infusion. In further fermentation, the flask was placed in a dark and clean environment at room temperature. Kombucha beverages were divided into four groups, including those fermented with *Rhodiola rosea* residue, without *Rhodiola rosea* residue, with *Salvia miltiorrhiza* residue, and without *Salvia miltiorrhiza* residue. Each group had three parallel samples. On Day 0, 3, 6, 9, 12, and 15, samples treated with a 0.22 μm syringe filter were used to assess the antioxidant activities, total polyphenol contents, and concentration of bioactive compounds.

### 3.3. Assessment of Antioxidant Activities and Total Polyphenol Contents

Trolox equivalent antioxidant capacity (TEAC) and ferric-reducing antioxidant power (FRAP) tests were used to assess the antioxidant activities based on previous studies [26,38].

For the TEAC test, 2,2′-azino-bis (3-ethylbenzothiazoline-6-sulfonate) positive free radical (ABTS^•+^) stock solution was prepared with 2.45 mmol/L potassium persulfate (K_2_S_2_O_8_) solution and 7 mmol/L ABTS solution at a ratio of 1:1 (*v*/*v*), which needed 16 h incubation in the dark. After incubation, the stock solution was diluted with distilled water to the absorbance of 0.71 ± 0.05 at 734 nm. The 100 μL diluted sample was mixed with 3.8 mL diluted ABTS^•+^ solution and then incubated in the dark for 6 min. Finally, we tested the absorbance of the mixture at 734 nm, and the data were represented in mmol Trolox/L.

For the FRAP test, FRAP solution was prepared by mixing 20 mmol/L ferric chloride (FeCl_3_) solution, 10 mmol/L 2,4,6-tri(2-pyridyl)-1,3,5-triazine (TPTZ) solution, and 300 mmol/L sodium acetate-acetic acid buffer in a volume ratio of 1:1:10, and then was put into the 37 °C water bath. A volume of 100 μL of diluted sample was mixed with 3 mL of FRAP solution and incubated at room temperature for 4 min. Lastly, we tested the absorbance of the mixture at 593 nm, and the data were represented in mmol Fe^2+^/L.

Total phenolic contents (TPC) were tested by the Folin–Ciocalteu method [39]. A volume of 500 μL of the diluted sample was mixed with 2.5 mL 0.2 mol/L Folin–Ciocalteu phenol reagent and then incubated in the dark for 4 min. After that, 2 mL 75 g/L sodium carbonate (Na_2_CO_3_) solution was added and further reacted in the dark for 2 h. In the end, the absorbance of the mixture was tested at 760 nm, and the data were represented in mg gallic acid equivalent (GAE)/L.

### 3.4. HPLC Analysis of Bioactive Compounds in Kombucha Beverages

A Waters 2695 HPLC, coupled with photodiode array detector (PAD) and Agilent Zorbax Eclipse XDB-C18 column (250 mm × 4.6 mm, 5 μm), was used to determine bioactive compounds. The column thermostat was set at 35 °C and sample thermostat was set at 4 °C. The mobile phase was the following gradient of solvent A (methanol) and solvent B (0.1% *v*/*v* formic acid in distilled water): 0–10 min (2–17% A); 10–15 min (17–19% A); 15–20 min (19–22% A); 20–40 min (22–47% A); 40–50 min (47–50% A); 50–60 min (50–58% A); 60–70 min (58–2% A); and 70–75 min (2% A) at a flow rate of 0.8 mL/min. The qualitative analysis was based on the retention time and UV–Vis spectrum compared with those of standard compounds. The quantitative analysis was performed by using a standard curve with the peak area under the maximum absorbance wavelength.

### 3.5. Sensory Analysis

The sensory analysis was conducted using a 9-point hedonic scale referring to previous studies [10,40]. Eight members (22–60 years old) with extensive experience, who have carried out sensory evaluation many times in our previous studies [10,23], from the Department of Nutrition, School of Public Health, Sun Yat-Sen University, independently rated the sensory properties of the kombucha beverages, considering the flavor, sourness, color, odor, and overall acceptability. The score of each parameter ranged from 1 (extreme dislike) to 9 (extreme like).

### 3.6. Statistical Analysis

All data were expressed as mean ± standard deviation and analyzed by one-way analysis of variance (ANOVA) followed by post hoc Tukey’s test. The correlation analysis was performed by Pearson’s product moment coefficient and heatmaps. The heatmaps were obtained by https://www.chiplot.online/, accessed on 17 June 2024. SPSS 25.0 statistical software (IBM Corp., Armonk, NY, USA) was used in statistical analysis. When *p* < 0.05, significant differences were considered to exist.

## 4. Conclusions

In this study, *Rhodiola rosea* and *Salvia miltiorrhiza* were used as the materials to prepare novel kombucha beverages for the first time, and these kombucha beverages showed strong antioxidant abilities and high total phenolic contents, especially the kombucha fermented with *Rhodiola rosea* residue. However, fermentation did not improve the antioxidant properties of the kombucha fermented without *Salvia miltiorrhiza* residue, despite increasing its TPC values. Moreover, fermentation with residue enhanced the concentrations of gallic acid, salidroside, and tyrosol in the *Rhodiola rosea* kombucha. The *Salvia miltiorrhiza* kombucha also contained various bioactive compounds, such as danshensu, salvianolic acid B, and salvianolic acid A. Furthermore, fermentation with residue did not affect the sensory properties of the kombucha beverages made from *Rhodiola rosea* and *Salvia miltiorrhiza*, and the kombucha fermented with *Rhodiola rosea* residue achieved the highest sensory scores among these kombucha beverages. Therefore, fermentation with *Rhodiola rosea* and *Salvia miltiorrhiza* can be good choices for preparing novel kombucha beverages.

## Figures and Tables

**Table 1:** The concentrations of compounds in *Rhodiola rosea* kombucha beverages fermented with or without residue.

Compound (mg/L)	Kombucha Beverage Type	Day 0	Day 3	Day 6	Day 9	Day 12	Day 15
Gallic acid	Without residue	86.59 ± 4.29	82.16 ± 8.00	83.62 ± 8.26	83.71 ± 6.56	87.47 ± 8.40	90.40 ± 6.73
	With residue	90.53 ± 3.84	152.43 ± 5.20	153.13 ± 7.68	155.65 ± 5.72	155.29 ± 7.84	157.29 ± 7.95
Salidroside	Without residue	44.50 ± 2.40	45.38 ± 2.99	45.35 ± 2.22	43.68 ± 2.50	40.20 ± 7.82	40.76 ± 4.09
	With residue	46.21 ± 2.78	94.70 ± 4.06	101.80 ± 4.66	109.84 ± 8.66	106.00 ± 4.80	105.08 ± 4.32
Tyrosol	Without residue	23.12 ± 2.28	24.13 ± 4.17	23.61 ± 3.83	26.33 ± 3.80	27.26 ± 4.62	24.73 ± 3.47
	With residue	24.57 ± 2.12	44.45 ± 3.21	53.36 ± 4.15	66.68 ± 3.96	54.41 ± 3.12	42.64 ± 4.40
EGCG	Without residue	36.81 ± 0.92	37.19 ± 0.75	37.66 ± 0.98	38.09 ± 1.15	39.05 ± 0.20	38.40 ± 1.17
	With residue	37.10 ± 0.93	29.41 ± 1.60	22.25 ± 0.77	21.02 ± 0.43	20.46 ± 0.26	19.97 ± 0.65

Abbreviation: EGCG, epigallocatechin gallate.

**Table 2:** The concentrations of compounds in *Salvia miltiorrhiza* beverages fermented with or without residue.

Compound (mg/L)	Kombucha Beverage Type	Day 0	Day 3	Day 6	Day 9	Day 12	Day 15
Danshensu	Without residue	7.72 ± 0.49	7.58 ± 0.48	7.79 ± 0.64	7.67 ± 0.56	7.77 ± 0.51	7.74 ± 0.52
	With residue	7.83 ± 0.26	10.32 ± 0.29	10.73 ± 0.28	9.77 ± 0.28	9.79 ± 0.29	9.70 ± 0.15
Salvianolic acid B	Without residue	191.58 ± 6.94	199.31 ± 3.99	189.57 ± 6.93	159.53 ± 4.64	149.21 ± 5.49	131.84 ± 6.90
	With residue	194.47 ± 7.65	334.77 ± 8.91	227.84 ± 5.68	187.97 ± 5.89	161.47 ± 6.21	134.55 ± 7.75
Salvianolic acid A	Without residue	2.89 ± 0.07	2.93 ± 0.05	2.71 ± 0.04	2.53 ± 0.09	2.41 ± 0.11	2.39 ± 0.18
	With residue	2.92 ± 0.08	4.19 ± 0.14	3.01 ± 0.02	2.54 ± 0.15	2.43 ± 0.15	2.41 ± 0.14

## Data Availability

The data are contained within the article.
